# Synthesis and Characterization of Biodegradable Poly(butyl cyanoacrylate) for Drug Delivery Applications

**DOI:** 10.3390/polym14050998

**Published:** 2022-02-28

**Authors:** Benjamin-Luca Keller, Claudia A. Lohmann, Samuel O. Kyeremateng, Gert Fricker

**Affiliations:** 1 AbbVie Deutschland GmbH & Co. KG, Knollstrasse, 67061 Ludwigshafen, Germany; samuel.kyeremateng@abbvie.com; 2 Waters Corporation, 34 Maple Street, Milford, MA 01757, USA; 3 Institute of Pharmacy and Molecular Biotechnology, University of Heidelberg, Im Neuenheimer Feld 329, 69120 Heidelberg, Germany; gert.fricker@uni-hd.de

**Keywords:** drug delivery, PBCA, molecular weights, biodegradable polymers, NMR, Advanced Polymer Chromatography™

## Abstract

Poly(butyl cyanoacrylate) (PBCA) is a biodegradable and biocompatible homopolymer which is used as a carrier matrix for drug delivery systems in the pharmaceutical industry. Typically, polymerization is carried out under aqueous conditions and results in molecular weights are mostly lower than 3000 g/mol due to the instability of the high molecular weight PBCA. However, the stability of polymer excipients is a major prerequisite for drug product development in the pharmaceutical industry. In this work, a reliable polymer synthesis strategy for PBCA was designed to control the molecular weight in a nonaqueous polymerization environment. The anionic polymerization process and the impact of key synthesis parameters were investigated. The results confirmed that the previously postulated depolymerization–repolymerization process (DPRP) in the literature can be used to tailor the molecular weight of PBCA. The amount of sodium methoxide present during the polymerization proved to be the key parameter to control the DPRP and the molecular weight as desired. In addition, it was discovered that end-capping the PBCA chain suppressed the DPRP and prevented monomer release by depriving the PBCA of its living character. Thus, neat PBCA polymer with varying molecular weights determined by Advanced Polymer Chromatography™ as well as end-capped PBCA were synthesized, and the improvement of the chemical and shelf-life stability were confirmed using NMR.

## 1. Introduction

Over the past few decades, nanomedicine has become a promising drug delivery strategy which provides solutions to increase drug substance concentrations at the site of action. The term nanomedicine is associated with the approach to target specific sites of action within a patient with drug-loaded nanoparticles (NP). These include polymeric NP, metal or metal oxide NP, nanocrystals, carbon-based NP, protein NP, lipid NP and liposomes [1,2,3,4,5,6,7,8]. Polymer-based NP have been known and investigated for the past four decades. In particular, polymeric NP comprised of biodegradable polymers derived from synthetic or natural polymers offer a promising approach for targeted drug delivery. Commonly used biodegradable and biocompatible polymers for polymeric NP design include poly(alkyl cyanoacrylates) (PACA) [9]. In 1979, Couvreur et al. reported the generation and characterization of PACA-based NP for the first time [10]. Since then, PACA NP have been extensively studied and frequently published [11,12]. Different PACA types with varying side alkyl groups up to C8 find use for NP production [12]. In the pharmaceutical field, poly(butyl cyanoacrylate) (PBCA) is a common representative of the PACA family, frequently studied as a drug delivery system to target cancer and/or pass the blood–brain barrier [11,13]. PBCA is a homopolymer composed of *n*-butyl-2-cyanoacrylate (BCA) monomer units. The monomer is successfully applied as a biomedical tissue adhesive due to the fast polymerization mechanism [14]. However, the lack of commercially available PBCA bears a challenge for the production of PBCA-based drug delivery systems. Thus, PBCA must be synthesized from the monomer prior to NP production. This distinguishes PBCA NP considerably from other polymeric NP systems (e.g., poly(lactic-co-glycolide) or poly(caprolactone)), where the polymer matrices are commercially available.

PBCA can generally be considered a pharmaceutical excipient; therefore, it has to meet the requirements regarding safety, functionality and quality [15,16]. The lack of commercially available PBCA has so far been compensated by applying BCA polymerization-based NP production methods where the in situ formation of polymer and nanoparticles take place simultaneously [11,12]. Within this approach, the anionic living polymerization is the predominant polymerization method for the synthesis of PBCA. The anionic polymerization of PBCA and its mechanism was intensively studied by Pepper et al. [17]. Although this polymerization-based method is mainly used to generate PBCA NP in the pharmaceutical field, the one-step process has two main drawbacks:

1. The characterization of PBCA as a defined pharmaceutical excipient to ensure quality prior to the NP formation is not possible;
2. The solvent-dependent molecular weight (MW) control insufficiency of PCBA during polymerization.

Within the pharmaceutical field, BCA is usually polymerized under aqueous conditions. In an aqueous medium, the formation of oligomeric units is favored, yielding a MW range of 1000–3000 g/mol [18]. Based on the literature, no polymeric NP-based delivery systems composed of PBCA exceed a MW of 3000 g/mol. However, in the field of polymeric NP, PBCA with higher MW is of great interest, since it was shown that the MW of biodegradable polymers can influence the physicochemical properties of the NP and thereby impact their performance after administration [19,20,21].

**Figure 1:**
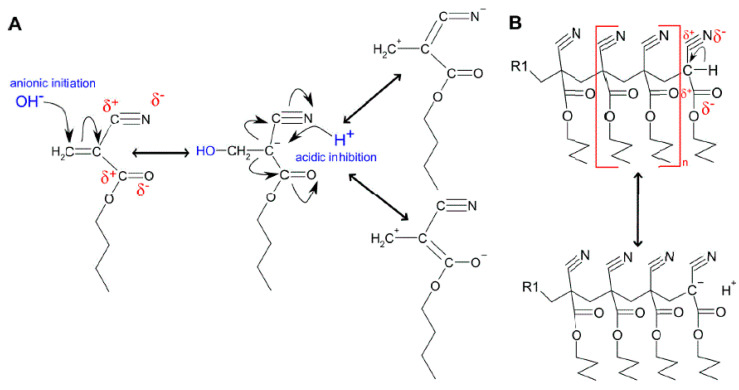
(**A**) Chemical structure of BCA, including possible resonance structures; (**B**) Chemical structures of PBCA and the origin of the C-H acidity appearing on the chain terminus of the polymer.

The reason for the insufficient polymerization control in an aqueous environment can be attributed to the high reactivity of the monomer’s chemical structure. The β-carbonyl-like structure of alkyl cyanoacrylates has a preferred tendency to form carbanions which undergo resonance stabilization induced by the strong electronegativity of the nitrile and carbonyl group (Figure 1A). This potent electron-withdrawing tendency increases the terminal C-H acidity as the chain length of the polymer increases (Figure 1B). Carbanion stabilization lends an exceptionally high reactivity to the monomer. The C-H acidity contributes to the living character of a PBCA molecule, enabling the polymer chain to restructure depending on the surrounding environment [18].

However, Pepper et al. showed that it is also possible to perform a living polymerization of PBCA in anhydrous THF as a polymerization medium [17,22]. Trace amounts of anions and weak bases such as hydroxides, alcohols, amines, etc., are sufficient to initiate the polymerization of PBCA. Furthermore, the researchers discovered a polymerization-inhibiting effect of strong acids that could also retard the polymerization of BCA [23,24]. The inhibition is caused by an excess of protons neutralizing the number of basic molecules in the reaction mixture as well as carbanion formation by covalently bonding to the α-carbon. Other possible polymerization mechanisms for PBCA are zwitterionic and radical polymerization. These are rarely performed in the pharmaceutical industry [25,26,27] because anionic polymerization always occurs as a side reaction.

Putting a strong focus on developing a reliable synthesis process for PBCA as an excipient calls for separating the synthesis process from the nanoparticle formation process in order to independently control the MW of PBCA. Therefore, in this work, the anionic polymerization mechanism of PBCA was studied and key polymerization process parameters were investigated and varied. The evolution of the polymerization process was monitored by Advanced Polymer Chromatography™ (APC™), determining the MW of the polymer at different stages of the polymerization process. APC™ is an UPLC-like instrument that is utilized for size-based separations, offering the unique capability of high-resolution analysis. Due to its ability to resolve low MW species down to the monomer level, APC™ proves to be a valuable tool for analyzing the changes during polymerization. The short runtimes of a single measurement also allow for a high sample throughput. NMR was used to verify the chemical structure of PBCA and monitor the postsynthesis end-capping functionalization of PBCA.

The overall aim of this work was to control and optimize the anionic polymerization process for PBCA to allow tailoring of the polymer MW. Keeping in mind that PBCA can potentially be applied as a pharmaceutical excipient for drug delivery, we further investigated the MW effect on the chemical stability of PBCA and end-capped PBCA.

## 2. Materials and Methods

### 2.1. Chemicals

Indermil (*n*-butyl cyanoacrylate monomer, BCA) was obtained from Henkel (Düsseldorf, Germany). AcroSeal™ tetrahydrofuran (THF) 99.5% extra dry was purchased from Acros Organics (Geel, Belgium) and trifluoroacetic acid (TFA) was purchased from Omnilab (Bremen, Germany). Sodium methoxide (NaOMe) powder, deuterated chloroform (CDCl_3_) and ethyl 2-(bromomethyl) acrylate (EBMA) were obtained from Sigma-Aldrich (Taufkirchen, Germany). A total of 0.1 M hydrochloric acid (HCl), chloroform (HPLC grade), methanol (HPLC grade), acetonitrile (HPLC grade) and HPLC-grade water were purchased from Merck (Darmstadt, Germany).

### 2.2. Characterization Techniques

#### 2.2.1. Advanced Polymer Chromatography™ (APC™)

ACQUITY™ APC™ from Waters Corporation (Milford, MA, USA) was used with a column bank consisting of an ACQUITY™ BEH XT 200 (4.6 mm × 150 mm, 2.5 μm) connected to two ACQUITY™ BEH XT 45 (4.6 mm × 150 mm, 1.7 μm) in series at 40 °C. THF was used as a mobile phase at a flow rate of 1 mL/min. Refractive index was used as a detector for the chromatograms. Conventional calibration was conducted with polystyrene standards. MW calculations were conducted with Empower™ with GPC option. Samples were dissolved in THF to make a final concentration of 5 mg/mL and 10 µL of the solution injected for the measurements.

#### 2.2.2. NMR

The 600 MHz ^1^H and 150 MHz ^13^C NMR spectra were obtained with a Bruker Avance™ system (Karlsruhe, Germany). Approximately 5 mg of samples were dissolved in 600 μL deuterated chloroform (CDCl_3_) for the measurements.

### 2.3. Synthesis

#### 2.3.1. Anionic Polymerization in THF

TFA diluted in 5 mL of THF was mixed with BCA in 20 mL scintillation vials. A total 120 µL of an aqueous solution of NaOMe was added under continuous magnetic stirring according to Table 1. Stirring at room temperature was continued for 180 min. Within this period, samples were taken at different time points and the polymerization was quenched immediately by precipitation in 0.1 M HCl (pH 1). The precipitates were frozen on dry ice and then lyophilized in an Alpha LSC 2–4 freeze dryer from Martin Christ (Osterode am Harz, Germany). Primary drying in the freeze dryer was performed for 12 h at 0.37 mbar with a shelf temperature of 0 °C. Secondary drying was done for 1 h at 0.01 mbar with a shelf temperature of 10 °C.

#### 2.3.2. PBCA End-Capping with EBMA in THF

A total of 2 mM TFA diluted in 5 mL THF was mixed with a 326 mM BCA solution under continuous magnetic stirring. The polymerization was started by adding 120 µL of an aqueous 8 mM or 80 mM NaOMe solution. Stirring was continued for 3 h. An amount of 2.5 mL of a 5 mM or 25 mM EBMA solution in THF was mixed with 2.5 mL of the PBCA polymerization mixture. The EBMA–PBCA mixture was stirred for 1 h. The EBMA-terminated PBCA (tPBCA) was precipitated in 0.1 M HCl (pH 1), purified by filtration and washed with deionized water to remove excess EBMA and lyophilized as described in Section 2.3.1.

#### 2.3.3. Polymer Stability in Solution

Approximately 5 mg polymer was dissolved in 600 μL deuterated chloroform and stored in a closed glass vial for seven weeks at room temperature. A 600 MHz ^1^H NMR spectrum was recorded each week.

## 3. Results

### 3.1. Polymerization Process Monitoring by APC™

Following Ryan et al.’s, approach, anionic polymerization was performed in THF [22]. The polymerization design comprised of reacting varying amounts of NaOMe, BCA and TFA. The evaluated polymerization conditions are reported in Table 1. The APC™ was used to monitor the evolution of the polymerization process and to investigate the impact of the polymerization condition on the MW of the target PBCA.

As described in Section 2, the BCA was diluted in THF containing TFA, which was used as a polymerization-retarding component. In general, the literature reports acids as polymerization-inhibiting components for the anionic polymerization of BCA [19,24]. Protons delivered by acids occupy the C-H acidic unit on the BCA structure. Consequently, carbanion formation is prevented and, therewith, the polymerization initiation. Costa et al. distinguished between the “polymerization inhibition” induced by strong acids (such as H_2_SO_4_ or CF_3_SO_3_H) and the “polymerization retardations periods” induced by weaker acids (such as ClCH_2_COOH) [24]. Thus, the presence of TFA prevented the spontaneous polymerization in the solvent THF before an initiator was added to the BCA–THF mixture. The presence of an acid is necessary because BCA has the tendency to self-initiate spontaneous polymerization, particularly in polar solvents such as THF [26].

**Figure 2:**
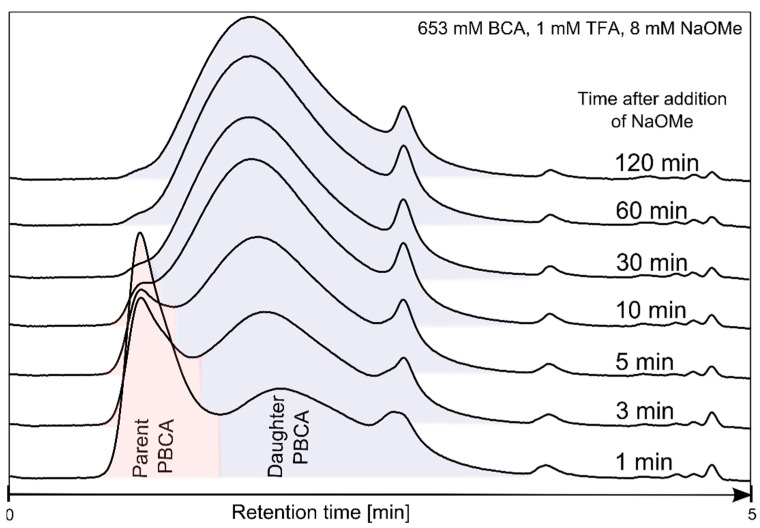
Time-dependent APC™ chromatograms monitoring the polymerization of PBCA following NaOMe addition.

The addition of different NaOMe amounts as the polymerization initiator led to the formation of mainly high molecular weight (HMW) PBCA, referred to as the “parent peak”, within a minute, as illustrated in Figure 2. Alongside the main HMW population is the low molecular weight (LMW) population, referred to as “daughter peak”, as well as a minor population at high retention times. Figure 2 also depicts the time-dependent emergence or growth of the “daughter peak” at the expense of the “parent peak” until all of the latter is consumed.

**Figure 3:**
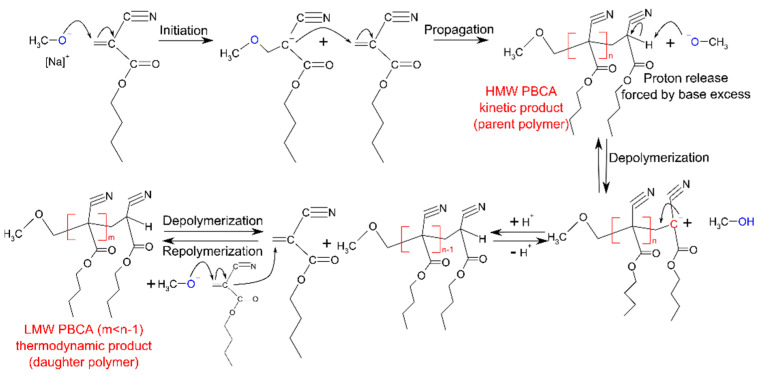
Initiation, propagation and depolymerization–repolymerization steps involved during anionic polymerization of BCA initiated by NaOMe in THF.

This indicates that the initial HMW PBCA underwent depolymerization, exhibited as decrease in the peak intensity and area. Thus, the depolymerization of the parent polymer was accompanied by a simultaneous repolymerization of the unzipped monomer. The distinctive formation, as well as the complete degeneration of the parent PBCA, was strongly dependent on selected polymerization conditions. The restructuring of PBCA from parent to daughter polymer, or the depolymerization–repolymerization process (DPRP), and the subsequent MW changes and relative peak areas are listed in Table 2. Similar observations were first reported by Ryan et al. [22]. The mechanism of the postulated DPRP is displayed in Figure 3.

**Figure 4:**
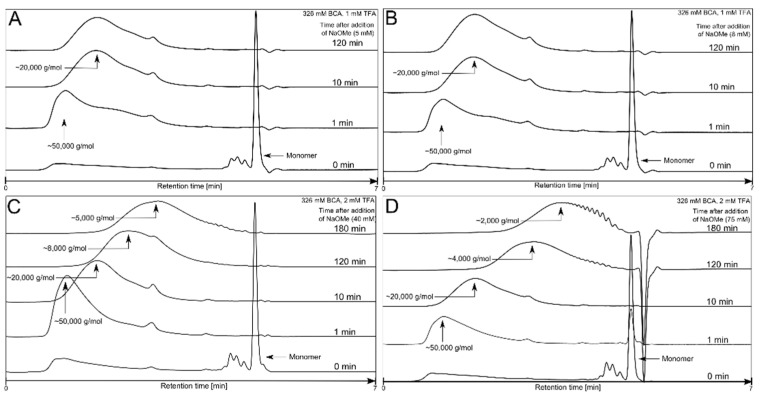
APC™ chromatogram of PBCA at different polymerization time points obtained with 5 mM NaOMe (**A**), 8 mM NaOMe (**B**), 40 mM NaOMe (**C**) and 75 mM NaOMe (**D**).

From a postulated mechanistic standpoint, the initial and rate determining step of the DPRP process is the proton liberation from the terminal unit in the polymer chain [22,28]. This proton release induces monomer unzipping from the chain terminus. Ryan et al. concluded from their studies that the excess base can facilitate the proton release and subsequently induce the formation of the LMW daughter PBCA [29]. The excess base forced the polymerization towards a thermodynamic product by deprotonation of the terminal polymer unit and thus reduced the activation energy of the rate-determining step. The researchers described the parent polymer as a kinetic product and the daughter polymer as a thermodynamic product. However, many of the investigated DPRP process conditions in this work resulted in a fluent transition from HMW to LMW PBCA without the appearance of two clearly separated parent and daughter fractions in the APC™ chromatogram, as can be seen in Figure 4. These transitions, along with the MW changes, appeared as a peak shift in the chromatogram from small retention times resembling HMW parent fractions to high retention times for the LMW daughter fractions.

**Figure 5:**
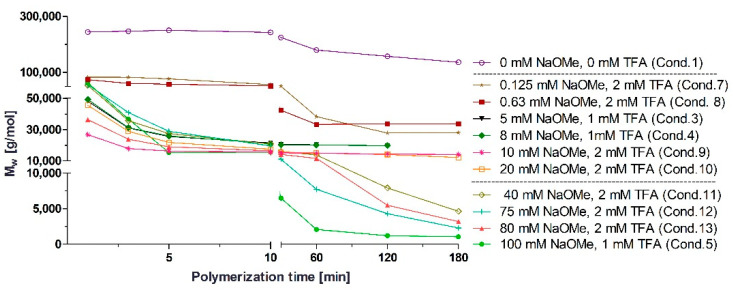
MW changes of PBCA during the anionic polymerization process induced by different NaOMe amounts (0–100 mM). A total of 326 mM BCA was used in all plotted conditions. MW was determined by APC™.

**Figure 6:**
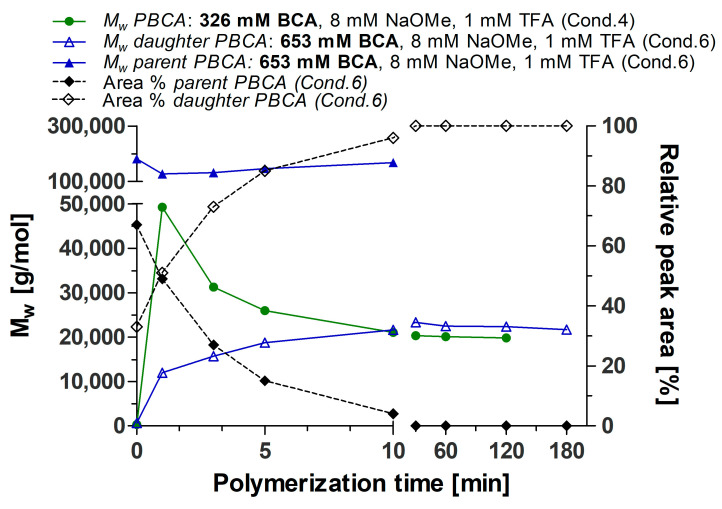
Influence of BCA amount on MW changes during the polymerization process. Comparison between 326 mM BCA and 653 mM BCA, both with 1 mM TFA and 8 mM NaOMe. MW was determined by APC™.

The results in Figure 5 and Figure 6 indicate that the DPRP of PBCA is affected by the amounts of NaOMe, BCA and TFA. NaOMe at varying amounts had the most profound impact on the DPRP. With increasing amounts of NaOMe, the MW decreased significantly within 180 min of polymerization. The trend of decreasing MW dependent on the NaOMe amount was observed in all polymerization conditions, as detailed in Figure 5.

The outcome of the trend plots (Figure 5 and Figure 6) clearly supports previous studies reporting that the DPRP can be influenced by basic molecules such as TBOH or NaOMe [22,30,31]. The results demonstrate that the effect on the PBCA MW depends on the NaOMe addition and supports the postulated reaction mechanism of the DPRP in Figure 3. Generally, it can be assumed that any base which can facilitate the proton release will equally reduce the activation energy and consequently force the DPRP towards LMW. Furthermore, different amounts of monomer with fixed amounts of TFA and NaOMe affected the polymerization process. An overall trend plot displaying the MW change induced by varying amounts of BCA is depicted in Figure 6. Polymerization with 326 mM BCA, 8 mM NaOMe and 1 mM TFA led to a fluent PBCA transition without the occurrence of a parent PBCA fraction.

In contrast, increasing the BCA amount (653 mM) decelerated the DPRP, which led to the distinct formation of parent PBCA and daughter PBCA fractions during the polymerization. It can be assumed that a larger monomer amount (653 mM) generated a larger number of HMW PBCA molecules. Hence, under similar conditions, more time is required to depolymerize the large number of HMW fractions compared to the polymerization with 326 mM BCA. Consequently, an increased amount of HMW PBCA molecules will extend the time for the total HMW PBCA consumption and therewith increase the transformation time of HMW PBCA to LMW PBCA. In this case, the HMW PBCA consumption delay prevented a fluent transition and led to the appearance of both fractions in the APC™ chromatogram. However, the final MW is similar (20,000 g/mol) in both the 326 mM and 653 mM BCA trials because the key factor for the final MW at the end of the polymerization is the NaOMe amount, which was kept constant at 8 mM (Figure 6).

### 3.2. NMR

#### 3.2.1. PBCA Structure Elucidation

**Figure 7:**
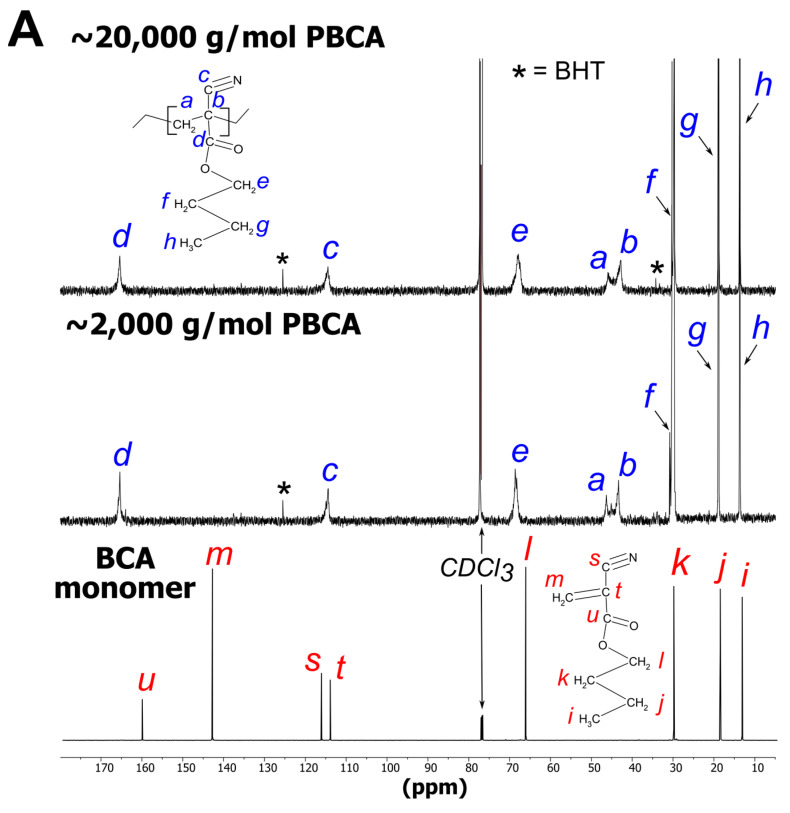

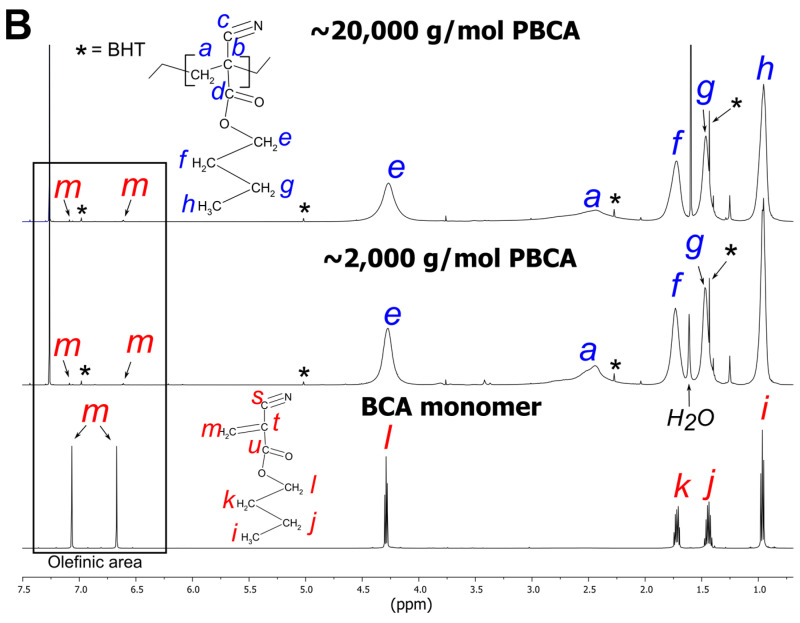
(**A**) The 150 MHz ^13^C NMR spectra of 2000 g/mol PBCA, 20,000 g/mol PBCA and BCA monomer with molecular structure peak assignments; (**B**) The 600 MHz ^1^H NMR spectra of 2000 g/mol PCBA, 20,000 g/mol PBCA and monomer with molecular structure peak assignments. Signals associated with butylated hydroxytoluene (BHT) impurity are marked as *.

Two PBCA batches with different MW (2000 g/mol and 20,000 g/mol) were analyzed by ^13^C and ^1^H NMR to verify the structure (Figure 7). Additionally, ^1^H and ^13^C spectra of the BCA monomer were recorded to identify potential residual monomer units. Both NMR measurements successfully confirmed the PBCA structure [30,32,33,34,35]. Differences between the 2000 g/mol and 20,000 g/mol PBCA batches in the ^13^C and ^1^H spectra appeared to be negligible. Therefore, the NaOMe amounts (8 mM or 80 mM) added to control the MW did not affect the PBCA structure from a chemical perspective. Both spectra revealed some unexpected signals (marked as * in Figure 7) originating from butylated hydroxytoluene (BHT) that stems from the THF polymerization medium. The BHT-laced polymers are indicative of an insufficient purification process at this lab scale and may require optimization in case of scale up. Alternatively, unstabilized BHT-free THF can be utilized instead.

Signal band broadening in the spectra suggest PBCA to be an atactic polymer (Figure 7A). In addition, the ^1^H spectra of the 2000 g/mol and 20,000 g/mol PBCA batches showed very weak signals (m) in the olefinic area at 7.06 ppm and 6.62 ppm. Comparing the polymer spectra with the monomer spectrum confirmed that these olefinic signals originated from the monomeric vinyl group (m) (Figure 7B). Similar observations of minor amounts of residual monomer (m) in the ^1^H spectra in the polymer were also made by other research groups after the polymerization of the alkyl cyanoacrylates [33,36]. The residual monomer amount was negligible and only detectable due to the low detection limit of the ^1^H NMR measurement. In the ^13^C NMR measurements, it was not possible to detect the residual monomer due to the lower sensitivity.

#### 3.2.2. tPBCA Structure Elucidation

For the systematic investigation of the living polymer character of PBCA, and potentially improving the stability of the PBCA polymer, the C-H acidic end group was substituted by EBMA in a postpolymerization reaction, as described by Kohsaka et al. [37]. Briefly, the EBMA acts as an efficient terminator by attacking the living carbanion of PBCA on the vinylidene group of the EBMA, with the subsequent elimination of bromine to generate an unsaturated ester moiety at the polymer end chain. Consequently, the termination with EBMA deprives the PBCA chain of its living character.

**Figure 8:**
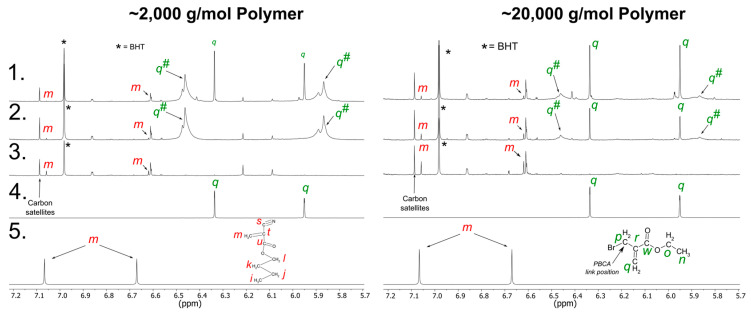
The 600 MHz ^1^H NMR spectra of the olefinic region. (**Left**): ~2000 g/mol tPBCA using 25 mM EBMA as terminator (**1**), ~2000 g/mol tPBCA using 5 mM EBMA (**2**), ~2000 g/mol PBCA as comparison (**3**), neat EBMA (**4**) and BCA monomer (**5**). (**Right**): ~20,000 g/mol tPBCA using 25 mM EBMA (**1**), ~20,000 g/mol tPBCA using 5 mM EBMA (**2**), ~20,000 g/mol PBCA as comparison (**3**), neat EBMA (**4**) and BCA monomer (**5**).

Two different MW batches of PBCA with a MW of 2000 g/mol and 20,000 g/mol were terminated at two EBMA concentrations, i.e., 25 mM or 5 mM. The structure of tPBCA was analyzed by NMR. The ^13^C and ^1^H spectra of tPBCA confirmed the successful linkage of the polymer and the terminal EBMA molecule (Figure 8).

Most importantly, the ^13^C and ^1^H spectra comparison of PBCA and tPBCA confirmed the integrity of the PBCA backbone structure after the addition of EBMA (Figure 8, ^13^C data not shown). The EBMA vinyl signal in the ^1^H spectrum was used to monitor the successful reaction between the EBMA and the polymer chain terminus. The different chemical shifts of the EBMA vinyl protons in the tPBCA spectra (q^#^) compared to the neat EBMA spectrum (q) confirmed that EBMA is chemically bonded to PBCA. The two proton signals of q^#^ were observed at a higher magnetic and lower magnetic field than the two protons of q, thus confirming the linkage between PBCA and EBMA. The observed band broadening of q^#^ in all the tPBCA samples can again be assigned to the atactic structure of the polymer. However, the q signal from the neat EBMA was still detectable in the ^13^C and ^1^H spectra of tPBCA for the 2000 g/mol and 20,000 g/mol PBCA batches reacted with 25 mM EBMA (Figure 8). Thus, the neat EBMA vinyl group signal in the tPBCA spectra proved the presence of unreacted EBMA molecules. Apparently, 25 mM EBMA bears an excess of the end-capping agent for both MW batches in this trial. A concentration of 5 mM EBMA reacted with the 20,000 g/mol PBCA batch also exhibited the neat EBMA vinyl group signal (q) in both ^13^C and ^1^H spectra. The 5 mM EBMA reacted with the 2000 g/mol batch showed no neat EBMA vinyl group signal (q) in the ^13^C and ^1^H tPBCA spectra, indicating a complete reaction between EBMA and the polymer.

These results confirm that the required amount of terminating agent depends on the polymer MW and, consequently, on the number of polymer chain termini. In an equal amount of polymer, the higher MW batch has a lesser number of chain termini compared to the lower MW batch. Consequently, the lower the PBCA MW, the higher the required terminator amount to react with all chain termini. The optimal EBMA amount for the termination of 2000 g/mol PBCA is lower than 25 mM and the optimal EBMA amount for the termination of 20,000 g/mol is lower than 5 mM. However, further studies are necessary to identify the exact EBMA amount required for a specific MW so to avoid residual unreacted EBMA molecules. In addition, the ^1^H spectra of all the tPBCA samples showed small amounts of residual BCA monomer (m), as described above for the PBCA polymers (Figure 8). The residual monomer probably originated from the NaOMe-induced monomer unzipping reaction during the DPRP prior to the addition of the end-capping agent EBMA. Based on the presence of the monomer in PBCA and tPBCA, a polymer stability study was conducted (see Section 3.2.3) to monitor the possible monomer release in PBCA and tPBCA, and to assess the stability, and thus the potential shelf-life of the polymers.

#### 3.2.3. Polymer Stability in Solution

**Figure 9:**
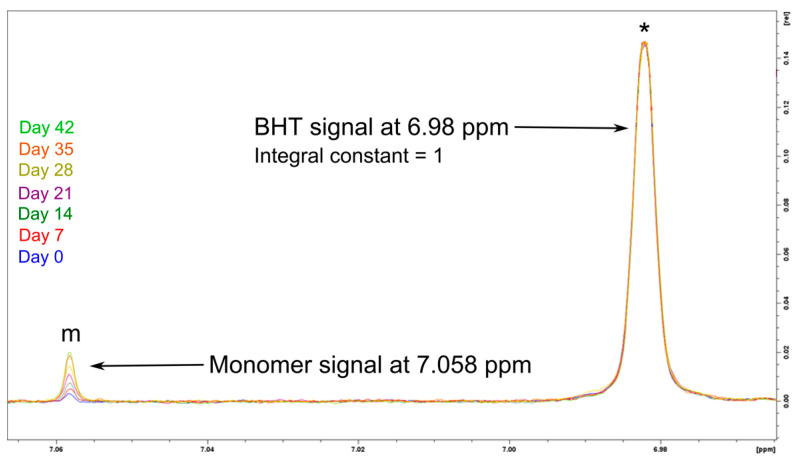
The ^1^H NMR spectrum section of the monomer (m) and BHT (*) signal in 2000 g/mol PBCA stored for seven weeks as solution in CDCl_3_. The BHT signal area (*) was constant during the storage, and consequently the integral value for BHT was assumed to be 1.

The stability of PBCA was monitored by measuring the increase of the monomer signal in the ^1^H NMR spectra. A signal increase over time would indicate potential chemical structural changes such as chain deconstruction or degradation. Two batches of neat PBCA and corresponding tPBCA having a MW of 2000 g/mol and 20,000 g/mol, respectively, were stored in CDCl_3_ solution for seven weeks. The ^1^H NMR spectra were recorded once a week, focusing on the monomer signal at 7.058 ppm. The relative monomer content was estimated using the BHT signal at 6.98 ppm as a constant reference (constant = 1). Figure 9 depicts the constant BHT signal in all measured samples during the monomer release study of the 2000 g/mol PCBA. The monomer signal marked ‘m’ increased steadily over the seven week period.

**Figure 10:**
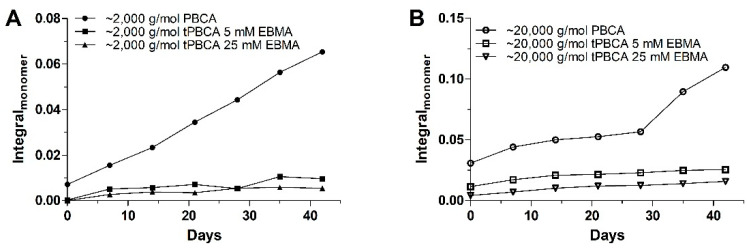
Monomer release monitored in ^1^H NMR measurements at 7.058 ppm (m) for PBCA and tPBCA. tPBCA was terminated using two different EBMA concentrations, 5 mM and 25 mM. PBCA and tPBCA having a MW of ~2000 g/mol (**A**) and ~20,000 g/mol (**B**) were stored for seven weeks in CDCl_3_ solution.

It can be assumed that the monomer residue originated from the NaOMe-derived monomer unzipping reaction (DPRP), resulting in the previously elucidated MW changes. However, the monomer release could also be a phenomenon induced by the living character of the PBCA chain progressing after the completion of the polymerization process. However, despite the living character of the PBCA as a polymer, it is essential that PBCA as a pharmaceutical excipient has a certain inherent chemical stability, especially to provide reasonable shelf-life. Thus, the end-capping of the chain termini using EBMA could potentially increase the shelf-life of PBCA as excipient by depriving PBCA of its living character and therewith preventing monomer release. The main observations made from the experimental results displayed in Figure 10 are:

1. The monomer release is indeed a progressive phenomenon for both PBCA batches, as the monomer amount clearly increased during storage;
2. The EBMA end-capping of the PBCA chains (tPBCA) inhibited the progression of the monomer release.

This proves that the PBCA chain is truly “alive”, thus also confirming the hypothesis of the monomer unzipping reaction. It also proves that the termination with EBMA deprived the PBCA chain of its living character because PBCA lost its C-H acidity at the end chain, and therefore the formation of carbanion was not possible. Consequently, the PBCA chain restructuring process (DPRP) was prevented by the end-capping with EBMA, which effectively suppressed the monomer release.

## 4. Conclusions

The process design of the anionic living polymerization of BCA in THF by varying the amounts of TFA, NaOMe and BCA demonstrated that the MW of PBCA was controllable within a range of 1000 g/mol to 200,000 g/mol. It turned out that the key factor for MW control was the added amount of NaOMe. Characterization by APC™ proved to be a good tool to monitor the polymerization process, while the NMR measurements confirmed the expected chemical structure. Both techniques also confirmed the postulated depolymerization–repolymerization behavior of PBCA. Stability studies on neat PBCA and EBMA-end-capped PBCA in solution were conducted over the course of seven weeks. Neat PBCA revealed a larger growth of free monomer over time due to the depolymerization, whereas the monomer growth was suppressed in the end-capped PBCA. The ongoing depolymerization of the polymer chain confirmed the living character of PBCA. The chemical instabilities of the neat polymer would cause a shelf-life reduction, thus impacting the quality of PBCA as an excipient. The present work demonstrates that low and high MW PBCA can be synthesized and end-capped as a possible means to increase the chemical stability and, hence, the shelf-life of the polymer. Increasing the shelf-life can potentially enable the diverse drug delivery applications of this biodegradable polymer.

## Figures and Tables

**Table 1:** Conditions during anionic polymerization in THF.

Condition	TFA (mM)	BCA (mM)	NaOMe (mM)
1	0.00	326	0
2	0.01	326	5
3	1.00	326	5
4	1.00	326	8
5	1.00	326	100
6	1.00	653	8
7	2.00	326	0.125
8	2.00	326	0.630
9	2.00	326	10
10	2.00	326	20
11	2.00	326	40
12	2.00	326	75
13	2.00	326	80

**Table 2:** MW and relative peak area of parent and daughter PBCA as determined by APC™ (653 mM BCA, 1 mM TFA and 8 mM NaOMe).

Time (min)	Parent PBCA		Daughter PBCA	
	MW (g/mol)	Relative Peak Area (%)	MW (g/mol)	Relative Peak Area (%)
0.5	181,488	67	645	33
1	127,605	49	12,023	51
3	131,730	27	15,751	73
5	145,970	15	18,775	85
10	168,314	4	21,623	96
30	-	0	23,328	100
60	-	0	22,496	100
120	-	0	22,388	100
180	-	0	21,713	100

## Data Availability

Restrictions apply to the availability of these data. Data was obtained from AbbVie and are not available without the permission of AbbVie.
